# Retraction Note: Detection of SARS-CoV-2 from patient fecal samples by whole genome sequencing

**DOI:** 10.1186/s13099-025-00711-6

**Published:** 2025-05-22

**Authors:** Andreas Papoutsis, Thomas Borody, Siba Dolai, Jordan Daniels, Skylar Steinberg, Brad Barrows, Sabine Hazan

**Affiliations:** 1 ProgenaBiome, Ventura, CA USA; 2 Centre for Digestive Diseases, Sydney, Australia; 3 Ventura Clinical Trials, Ventura, CA USA

**Retraction Note: Gut Pathogens (2021) 13:7**

10.1186/s13099-021-00398-5

The editors have retracted this article. After publication discrepancies between the registered protocol of the trial and the work reported in the article were noted and the authors were unable to provide documentary evidence of ethical approval for the changes made to the protocol. Concerns were raised regarding the accuracy of the stated study period as samples were collected prior to the registration of the study and collected after the study period had ended. Furthermore, a number of participants did not meet the stated inclusion criteria but were still included in the study. None of the authors have responded to any correspondence from the editor about this retraction.

